# Contrasting Metabolic Fingerprints and Seed Protein Profiles of *Cucurbita foetidissima* and *C. radicans* Fruits from Feral Plants Sampled in Central Mexico

**DOI:** 10.3390/plants10112451

**Published:** 2021-11-13

**Authors:** Claudia Mejía-Morales, Ramón Rodríguez-Macías, Eduardo Salcedo-Pérez, Juan Francisco Zamora-Natera, Fabián Alejandro Rodríguez-Zaragoza, Jorge Molina-Torres, John Paul Délano-Frier, Julia Zañudo-Hernández

**Affiliations:** 1 Departamento de Ecología, Centro Universitario de Ciencias Biológicas y Agropecuarias, Universidad de Guadalajara, Zapopan 44600, Mexico; claudia.mmorales@alumnos.udg.mx (C.M.-M.); fabian.rzaragoza@academicos.udg.mx (F.A.R.-Z.); 2 Departamento de Botánica y Zoología, Centro Universitario de Ciencias Biológicas y Agropecuarias, Universidad de Guadalajara, Zapopan 44600, Mexico; ramon.rmacias@academicos.udg.mx (R.R.-M.); eduardo.salcedo@academicos.udg.mx (E.S.-P.); juan.znatera@academicos.udg.mx (J.F.Z.-N.); 3 Departamento de Biotecnología y Bioquímica, Centro de Investigación y de Estudios Avanzados del Instituto Politécnico Nacional, Irapuato 36824, Mexico; jmolina@cinvestav.mx

**Keywords:** cucurbitacins, flavonoids, morphology, non-structural carbohydrates, neutral lipids, phenolic acids, proximal composition, raffinose family oligosaccharides

## Abstract

*Cucurbita foetidissima* and *C. radicans* are scarcely studied wild pumpkin species that grow in arid and semi-arid areas of Mexico and the United States. This study describes the morphological, proximal composition, metabolic finger-prints and seed protein profiles of *C. foetidissima* and *C. radicans* fruits collected in the wild during a one-year period in different locations of central-western Mexico. The results obtained complement the limited information concerning the fruit composition of *C. foetidissima* and greatly expand information in this respect regarding *C. radicans*. Morphology and proximal composition of their fruits varied significantly. Different metabolic fingerprints and seed protein profiles were detected between them and also with the chemical composition of domesticated *Cucurbita* fruits. The neutral lipids in seed, pulp and peels were rich in wax content and in unsaturated compounds, probably carotenoids and tocopherols, in addition to tri-, di- and mono-acylglycerols. The tri- and diacylglycerol profiles of their seed oils were different from commercial seed oils and between each other. They also showed unusual fatty acid compositions. Evidence of a possible alkaloid in the pulp and peel of both species was obtained in addition to several putative cucurbitacins. An abundance of phenolic acids was found in all fruit parts, whereas flavonoids were only detected in the peels. Unlike most cucurbits, globulins were not the main protein fraction in the seeds of *C. radicans*, whereas the non-structural carbohydrate and raffinose oligosaccharide content in their fruit parts was lower than in other wild cucurbit species. These results emphasize the significantly different chemical composition of these two marginally studied *Cucurbita* species, which was more discrepant in *C. radicans*, despite the notion regarding *C. foetidissima* as an aberrant species with no affinity to any other *Cucurbita* species.

## 1. Introduction

Mexico is a mega-biodiverse country reporting more than 26,000 identified endemic native vascular plant species, a high percentage of which are employed for medicinal, food and/ or ornamental purposes [1]. Notable progress has been made in the overall characterization of many of these plants. However, knowledge regarding several non-forest herbaceous plant species that have the potential to be exploited for commercial use, such as *Cucurbita foetidissima* Kunth and *C. radicans* is limited [2,3,4]. *Cucurbita foetidissima* is part of a group of perennial xerophyte species of this genus, together with *C. palmata*, *C. digitata* and *C. cilindrata* [2,4,5,6]. *Cucurbita radicans* and its closely related species, *C. pedatifolia*, are usually classified as semi-xerophytic due to their closer relationship to annual-mesophytic species, although this diversification has been questioned [7,8]. Moreover, the lack of properly documented morphological, ecological and genetic differences between them has cast doubt upon their classification as different species, despite their obvious differences in leaf morphology and shape, root size and growing habits [4,5]. It has been reported that *C. pedatifolia*, *C. radicans* and *C. foetidissima* have the closest relationship with domesticated cucurbit species [4].

*Cucurbita foetidissima* is an herbaceous plant commonly known as buffalo, fetid, stinking, wild, coyote, Missouri or prairie gourd, in addition to “calabacilla de burro” and “chilicote”. The wide-ranging territorial extension of *C. foetidissima* includes southwest and central regions of the United States and spreads to several northwestern and central states of Mexico [9]. This species grows in semi-arid locations positioned between 1600 and 2500 m above sea levels (m a.s.l.). The plants are perennial, highly polymorphic, xerophytic and hemicryptophytic, able to generate buds at or near the soil surface. They are also tolerant to high temperatures and to biotic stressors, the latter related, in part, to the accumulation of ribosome inhibiting proteins [10]. *Cucurbita radicans*, also known as “calabacilla” or coyote gourd, is endemic to the Mexican Neo-volcanic axis, extending from Zacatecas, Guanajuato and Estado de Mexico in central Mexico, to the western Jalisco, Michoacán and Nayarit states. Similar to *C. foetidissima*, it is found at an altitude range of 1750 to 2250 m a.s.l. [11]. It is considered to be an endangered species due to increasing urbanization, loss of habitat and agricultural expansion [12]. It does not have the unpleasant odor characteristic of *C. foetidissima*, although both species produce small, bitter-tasting fruits with few seeds that are larger, but less abundant, in *C. radicans* [11].

*Cucurbita foetidissima* plants can be frequently found in disturbed habitats such as roadsides, railroad tracks and wastelands, along with wild grasses and xerophyte shrubs [9,13]. Here, the environmental conditions usually differ from the natural surroundings in terms of plant species composition, moisture, temperature and nutrients [14]. These plants, that usually spread as creeping stems, can extend more than 200 m in a single 5-month growing season [2,3,15]. They have deep, fibrous roots that can reach 40 kg in weight [9,13,16,17,18]. This large root system can grow to a depth of 5 m to function as a water reservoir and a nitrogen scavenger [19]. It also represents an important source of carbon and energy stored as starch [9,20]. These properties, together with the suberization of the root peridermis that favors water retention, allow the survival of these plants in dry habitats or in sites where run-off water is the only source of humidity [3,20]. It requires a minimum of 150 mm of annual rainfall precipitation to survive, whereas 250 mm is indispensable for proper plant reproduction and fruit yield [17,20]. Their abundant root starch energy reserves compensate for the high sensitivity of this plant’s foliage to the cold temperatures that characterize many of its habitats during the winter, allowing for seasonal regrowth [14,18]. *Cucurbita radicans* is predominantly found as solitary plants growing among shrubs, or in grasslands and/or lowlands [12], frequently in association with woody leguminous species, such as *Prosopis* sp. and *Vachellia* sp., together with wild grasses and castor bean plants or within agave or maize plantations [11,21]. 

Both plants are employed for several traditional uses by indigenous people, although *Cucurbita radicans* not as widely as *C. foetidissima*. Their roots, fruits and/or seeds are diversely employed for the treatment of rashes, inflammations, stomach ailments, intestinal parasites, urinary tract infections and rheumatism, among others. They are also consumed as a porridge or beverage, and used for the elaboration of artisanal soaps that in some regions may be utilized for washing lamb wool prior to pigmentation [2,6,9,17]. However, *C. radicans* is also considered a noxious, invasive, weed and is frequently combatted as such [4,5,6].

*Cucurbita foetidissima* has long been considered to have a high potential for agro-industrial use. Some relevant examples are its extensive tap-root system which, being rich in starch content, may be used for human or animal nutrition [3,22]. This rich starch source has also been appraised for bio-fuel production or as a food supplement. The former is based on evidence showing that the root starch of *C. foetidissima* produced superior ethanol yields compared to corn or sorghum grains [16,17,19,23,24,25]. The seeds, known to reach yields that approximate 3000 kg/ha [18], contain high amounts of protein and superior-quality oil similar in composition to sunflower oil [2,3,17,19,26]. Conversely, *C. radicans* develops multiple small and tuberous ovoid roots rather than an extensive taproot system [13]. This root phenotype is believed by some workers to be a trait that may be beneficial for the development of drought-tolerant *Cucurbita* starch crops [27]. Moreover, a potentially large-scale pharmacological use of these species, already recognized by several rural populations of Mexico and the USA, stems principally from the abundance of saponins and cucurbitacins that characterize the bitter flesh of their fruits [28] and from further pharmacological properties recently found in the oil of cultivated *Cucurbita* subspecies [29].

However, the attractiveness of *C. foetidissima* as a potentially lucrative cash crop has been hampered by their highly inconsistent plant growth and fruit yields produced in response to fluctuating ambient conditions, predominantly in terms of solar radiation and rainfall levels, in addition to the presence of competing species [3,16,19]. Likewise, the very limited knowledge regarding the chemical composition and growth habits of *C. radicans* has obstructed its consideration for more extensive cultivation outside small pockets in the states of Jalisco, Michoacán and rural zones of Mexico City [4,5]. The morphological and genetic variability of *C. foetidissima* is also shared with cultivated *Cucurbita* plant populations. For example, the variability observed within *C. maxima* populations in Turkey was associated with key factors occurring during cultivation, harvest and storage [30]. Within this context, the present field study was performed in order gain a deeper knowledge of the chemical composition of *C. foetidissima* and *C. radicans* fruits field-collected in different localities of Mexico in the year 2018. The results obtained expand existing information regarding the chemical composition of *C. foetidissima* fruits. The latter was found to differ significantly from the practically unknown composition of *C. radicans* fruits, notwithstanding their close phylogenetic relationship [7,8]. The results also showed important differences with the fruit composition of cultivated *Cucurbita* species and suggested a great level of plasticity in the wild species that occurred in response to fluctuating ambient factors. The results from this study also offer an insight into unique chemical constituents present in the fruits of these highly adapted undomesticated plant species which, as mentioned above, have the potential to be employed for either food, industrial or biomedical purposes. 

The information provided is also valuable in the context of the potential use of these two cucurbit species for profitable agricultural programs in arid and semi-arid regions [16,22,31,32,33,34,35,36]. In this respect, *C. radicans* and *C. foetidissima* are considered as a vast source of genetic variability for the development of, for example, domesticated *Cucurbita* cultivars having increased resistance to (a)biotic stress, predominantly caused by drought, viral and fungal infection and insect infestation [37,38]. In principle, these could be generated utilizing the assumed capacity that all wild *Cucurbita* species have to cross with one or more other species in the genus [39,40], although crosses between secondary and tertiary wild *Cucurbita* resources, such as *C. radicans* and *C. foetidissima*, respectively, may require the use of elaborate breeding techniques [41]. The introduction of domestication traits into these wild species, including an increase in the size of fruits and a reduction in the bitter taste of their pulp, among others [42], could renew their attractiveness as a lucrative cash crop for semi-arid regions of Mexico. In this respect, it is relevant to consider that all Mexican semi-arid regions’ ecosystems are highly sensitive to climate variability. The projected global environmental changes predict that these regions will experience an increase in both mean maximum and mean minimum temperatures and an increment in the duration and severity of drought events [43]. The consequent reduction of water availability and of arable land will necessarily increase the demand for drought-tolerant crops. This critical change may renew the interest in the cultivation of drought-resistant plants, including the wild *Cucurbita* species herewith described, which could become part of national food security programs for these ecologically vulnerable habitats. 

## 2. Results

### 2.1. Morphological and Proximal Composition Variables 

The average results obtained from the measurement of morphological variables of *C. foetidissima and C. radicans* fruits sampled in the wild are shown in Table 1. Regarding *C. foetidissima*, they complement previous studies describing the morphological variability of fruits obtained from plants sampled in the wild in Arizona and elsewhere [3]. Several differences in morphological variables were found between fruits of *C. foetidissima* and *C. radicans*. The fruits of *C. foetidissima* were significantly heavier and longer and had a significant 3-fold higher number of seeds. Significant differences in the proximal composition of the seeds, peels and pulps of their respective fruits were also found (Table 2). Thus, seeds of *C. foetidissima* were richer in non-polar components, as defined by the ether soluble fraction, whereas those of *C. radicans* had a higher crude protein content. Peels of both fruits were highly fibrous, although several fiber components were significantly higher in those of *C. foetidissima* fruits. The pulp was rich in minerals and N-free extract, which were components that were significantly higher in *C. radicans* and *C. foetidissima* fruits, respectively. 

### 2.2. Neutral Lipids

**Figure 1:**
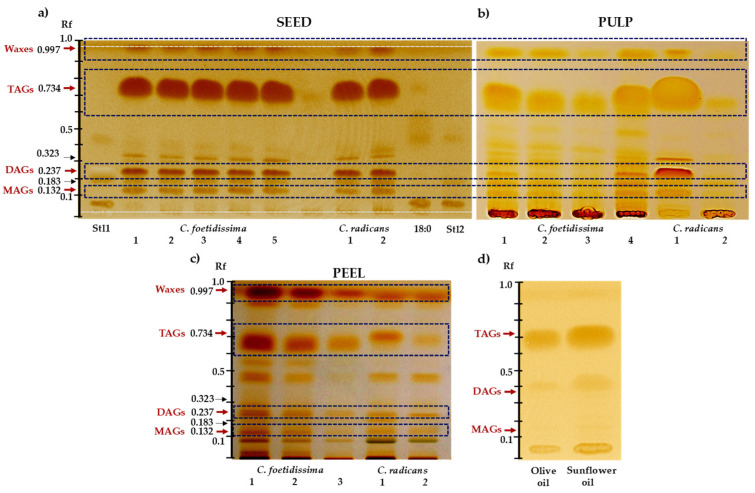
Neutral lipids profiles in fruits of wild *Cucurbita* species. Neutral lipid profiles of (**a**) seeds, (**b**) pulp and (**c**) peel of *C. foetidissima* and *C. radicans* fruits were obtained by HPTLC analysis. They were compared to (**d**) olive oil and sunflower seed oil, respectively and to steric acid (18:0) and two plant sterol standards (Stl1 and Stl2). The fruits were sampled in 2018 from plants growing in the wild in two different locations in the state of Jalisco, México: Vaquerías, municipality of Ojuelos (*C. foetidissima*) and San José de Gracia, municipality of Tepatitlán (*C. radicans*). The red and black arrows indicate the strongest signals obtained, corresponding to triacylglycerols (TAGs), diacylglycerols (DAGs), monoacylglycerols (MAGs), waxes and other unidentified compounds.

The HPTLC analysis of seed hexane extracts shown in Figure 1a indicated a predominance of triacylglycerides (TAGs). Weaker bands with Rf values corresponding to diacylglycerides (DAGs) and monoacylglycerides (MAGs) were also detected. TAG abundance appeared to be similar to that detected in olive and sunflower seed oils (Figure 1d), whereas MAGs and DAGs were clearly higher in the seed oils of both *Cucurbita* species examined. The neutral lipid composition observed was similar in seeds of the two *Cucurbita* species analyzed (Figure 1a). In contrast to the seed oils of olive and sunflower plants (Figure 1d), the content of waxes and other highly non-polar hexane-soluble contents was also higher. A strong signal at the baseline of the TLC plates was suggestive of an abundant fraction of polar lipids, such as phospholipids, in the seed oils of both *Cucurbita* species. The strong reaction with iodine fumes was indicative of high unsaturation levels. TLC data was in accordance with the MALDI-TOF analysis that indicated the presence of several DAGs and TAGs composed of unsaturated fatty acids in the seed oil of these cucurbit species (Table 3; Figure S1). The presence of some unidentified ions not present in common oils was suggestive of TAGs/DAGs having unusual composition. Differences were also observed between the DAGs and TAGs composition of the seed oils of *C. foetidissima* and *C. radicans*.

The neutral lipid composition of the *Cucurbita* fruit pulps (Figure 1b) was characterized by strong TAG bands in addition to other bands corresponding to several other non-polar and unsaturated compounds not detected in seed oils. The signal at the base line was stronger than in seeds, indicating the presence of phospholipids and perhaps other compounds, such as tetracyclic isoprenoids. Waxes were also very abundant. Also remarkable was that the pulp of the two *Cucurbita* species analyzed had unique non-polar lipid metabolic fingerprints. The neutral lipid content of the peels (Figure 1c) was also rich in iodine-reactive non-polar hexane-soluble compounds. Particularly abundant were bands with Rf values corresponding to waxes.

**Figure 2:**
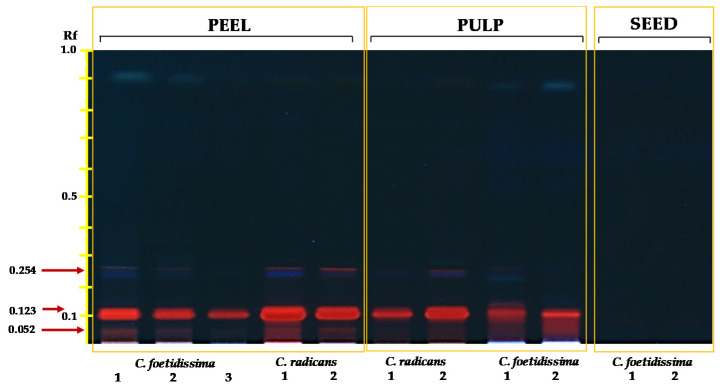
Fluorescent fruit hexane extract profiles of wild *Cucurbita* species. Hexane extract profiles of seeds, pulp and peel of *C. foetidissima* and *C. radicans* fruits were obtained by HPTLC analysis. The fruits were sampled in 2018 from plants growing in the wild in two different locations in the state of Jalisco, México: Vaquerías, municipality of Ojuelos (*C. foetidissima*), and San José de Gracia, municipality of Tepatitlán (*C. radicans*). The red arrows indicate the fluorescent signals obtained when the TLC plates were visualized under UV light (366 nm).

Naturally fluorescing bands with Rf values between 0.05 and 0.26 were also detected in hexane extracts of these fruits. Their mobility and fluorescence emission suggested the presence of unidentified alkaloids or, more probably, saponins (Figure 2). They were detected in the pulp and peel of both *Cucurbita* species analyzed. The bands appeared to be more intense in pulp and peel extracts obtained from *C. radicans* fruits

### 2.3. Saponins/Cucurbitacins 

**Figure 3:**
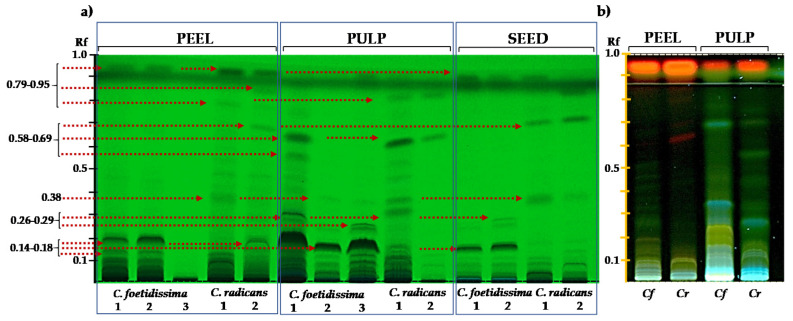
Saponin/cucurbitacin profiles of wild *Cucurbita* species. Saponin/cucurbitacin profiles of seeds, pulp and peel of *C. foetidissima* and *C. radicans* fruits were obtained by HPTLC analysis. The fruits were sampled in 2018 from plants growing in the wild in two different locations in the state of Jalisco, México: Vaquerías, municipality of Ojuelos (*C. foetidissima*) and San José de Gracia, municipality of Tepatitlán (*C. radicans*). In (**a**) the TLC-separation of ethanolic extracts was visualized under UV light at 254 nm. The arrows indicate bands whose Rf values corresponded to those of reported cucurbitacins. In (**b**) a similar TLC separation of pulp and peel methanol-chloroform extracts is shown when visualized under UV light (366 nm). *Cf* = *Cucurbita foetidissima*; *Cr* = *Cucurbita radicans*.

The saponin metabolic fingerprint was characterized by several bands in the fruit parts of the species examined (Figure 3). The peel was a rich source of these compounds. A marked difference between *C. foetidissima* and *C. radicans*, was that the former was composed almost exclusively of polar forms having Rf values ≤ 0.2, a pattern that suggested the presence of glycosylated cucurbitacin glycosides [44]. Although the putative glycosylated forms were also abundant in *C. radicans*, the presence of more mobile bands with higher Rf values were suggestive of the presence of free cucurbitacin aglycones as well. These compounds were detected in the three fruit parts analyzed; however, they were more abundant in the pulp and peel. A different abundance of these bands in the pulp of *C. radicans* fruits was also observed. Different band patterns were also detected in the seed extracts of *C. foetidissima* and *C. radicans*. Comparison with reported Rf values suggested the presence of various cucurbitacins and cucurbitacin glycosides in the fruit of these species (Table 4).

### 2.4. Phenolics 

**Figure 4:**
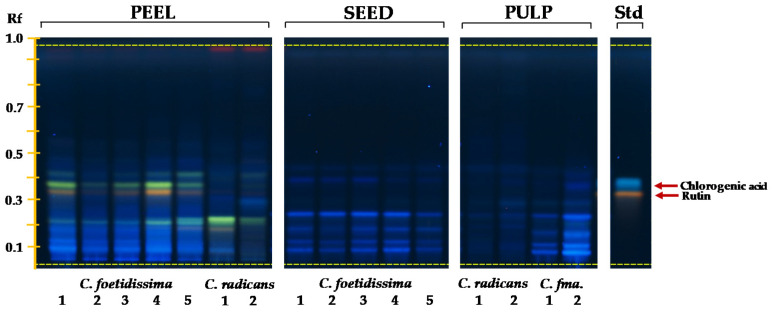
Phenolic compound profiles of fruits of wild *Cucurbita* species. Phenolic compound profiles of seeds, pulp and peel of *C. foetidissima* and *C. radicans* fruits were obtained by HPTLC analysis. The fruits were sampled in 2018 from plants growing in the wild in two different locations in the state of Jalisco, México: Vaquerías, municipality of Ojuelos (*C. foetidissima*), and San José de Gracia, municipality of Tepatitlán (*C. radicans*). The TLC-separation of methanolic extracts was visualized under UV light (366 nm) after derivatization with the *Natural Products* reagent. The red arrows indicate bands whose Rf values corresponded to those of reported standards (Std): Rutin and chlorogenic acid. *C. fma.* = *Cucurbita foetidissima*.

The use of the NP reagent permitted a better visualization of the phenol and flavonoid compounds (Figure 4). Peels were the fruit part having the most intense signal for phenolic compounds. This was more evident in peels of *C. foetidissima*, which were rich in a compound having similar characteristics, in terms of its orange–yellow color and Rf value, to the rutin standard. Other similarly pigmented bands but with lower Rf values were detected in the peels of *C. radicans* fruits. Bands having the characteristic blue coloration produced by phenolic acids or coumarins, which migrated within a 0.05-to-0.25 Rf range, were prevalent in the pulp of *C. foetidissima* fruits but not in those of *C. radicans*. The band-pattern in *C. foetidissima* seeds was similar to that of the pulp, but slightly less intense. 

### 2.5. Seed Proteins

**Figure 5:**
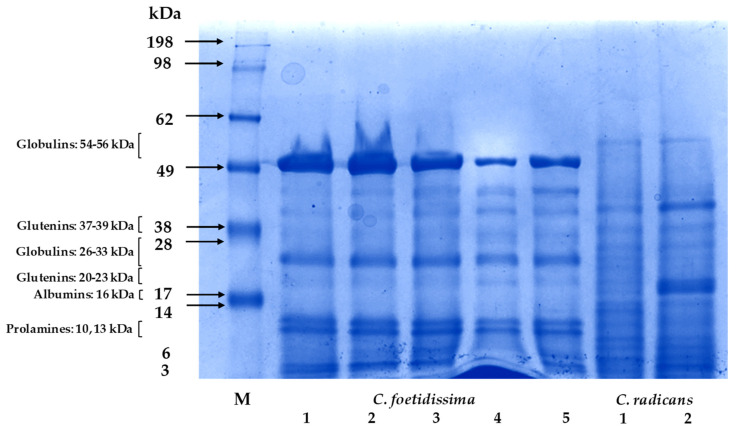
Seed protein profiles of wild *Cucurbita* species. The SDS-PAGE separation of seed proteins from *C. foetidissima* and *C. radicans* fruits is shown. The fruits were sampled in 2018 from plants growing in the wild in two different locations in the state of Jalisco, México: Vaquerías, municipality of Ojuelos (*C. foetidissima*), and San José de Gracia, municipality of Tepatitlán (*C. radicans*). The protein bands were visualized after incubation in a Coomasie Blue stain solution. The arrows indicate bands whose Rf values corresponded to seed proteins described in related *Cucurbita* species [45,46,47,48]. M = molecular mass protein markers, in kilo Daltons (kDA).

A difference in the type of proteins was present in the seeds of *C. foetidissima* and *C. radicans* (Figure 5). Intense bands that migrated in a zone corresponding to 10–13 kDa prolamines and 26 and 54–56 kDa globulines were abundant in seed proteins of *C. foetidissima* but practically absent in the seed proteins of *C. radicans*. In contrast, bands whose mass corresponded to glutelins (23 and 37–39 kDa) were richer in seed proteins of *C. radicans***.** A curious difference observed was that the 54–56 kDA protein bands of *C. foetidissima* (samples 1 to 3) were stronger in seed extracts obtained from plants whose roadside orientation to the West exposed them to shorter periods of solar radiation [21]. 

### 2.6. Non-Structural Carbohydrates (NSCs) and Raffinose Family Oligosaccharides (RFOs)

Glucose (Glc) and fructose (Fru) levels were the most abundant soluble NSCs detected in *C. foetidissima* and *C. radicans* fruits. This was particularly evident for Glc levels in pulp and peels of *C. foetidissima* (Table 5). Although present at much lower levels than Glc and Fru, sucrose (Suc) content was highest in the seeds, again significantly so in those of *C. foetidissima* fruits. A similar tendency was found for starch, with significantly higher levels detected in the pulp and peel of *C. foetidissima* fruits, respectively. As expected for oleaginous seeds, starch levels, although significantly higher in seeds of *C. foetidissima*, were much reduced compared to cereals and other seeds (Table 5). 

The RFO precursors myo-inositol (MI) and galactinol (GOL) were highest in pulp and peels. MI and GOL were significantly higher in the seeds and peels of *C. radicans*, and in seeds of *C. foetidissima*, respectively. Raffinose (Raf) and staquiose (Sta) RFOs were much more abundant in seeds (Table 6). Both RFOs were significantly higher in the seeds of *C. foetidissima* fruits. Also relevant was the detection of an unidentified RFO with a retention time similar to trehalose, mostly in the pulp of *C. foetidissima* fruits.

## 3. Discussion

This field study was performed in order to gain a deeper knowledge of the phytochemical composition of the fruits of two plant species that conform the group of 11 wild *Cucurbita* species that are found in extensive regions of Mexico [7]. The information generated was particularly relevant for *C. radicans*, a species endemic to México, considered by some to be endangered due to habitat loss and other relevant factors, for which very limited information is available regarding the chemical composition of its fruit [7,12,49].

With respect to *C. foetidissima*, the study was performed in communities located in the state of Jalisco, México, that were close to the locations of a related field study that included the nearby states of San Luis Potosí and Zacatecas [3]. The results obtained further complemented the knowledge concerning the growth habit and the quality and fruit yield of *C. foetidissima* plants that were most frequently found in disturbed habitats, including roadsides, where the environmental conditions tend to be significantly different from those in natural surroundings [8,14]. Although apparently very similar, several differences in the physiological and morphological characteristics of *C. foetidissima* and *C. radicans* fruits were found. Contrary to *C. foetidissima*, the fruits of the former species were less abundant, hidden behind dense wild-grass growth or rocks and mostly developed in the shade, many times attached to dried-up branches. They also had a very slow maturation rate (≥ 6 months) after being separated from the plants, a process that also led to changes in fruit pigmentation and to significant weight loss [21]. These characteristics were factors that hindered a more complete phytochemical characterization. They also strongly suggest that the apparently non-climacteric fruit maturation process in these two species is very different. As reported previously [11,25], the shape of their fruits was similarly globular, although *C. foetidissima* fruits were longer. They were also significantly heavier, having a significantly higher number of seeds per fruit, which were fleshier and coarser. Certain parameters, such as FFW, SN, W100S, LD and ED were also higher than those reported previously in fruits collected from plants sampled in the state of Arizona, USA [23,24]. On the other hand, the seeds of *C. radicans* fruits were significantly heavier. The differences observed between this and other studies could be tentatively attributed to: (i) different seasonal fluctuations in growth rate and in the prevalence of competing species, mostly for soil nitrogen, as determined by the contrasting ambient conditions present in each particular zone [19], and (ii) the phenotypic plasticity that characterizes the *Cucurbita genus* [30], and *C. foetidissima* in particular, the latter in terms of seed yield and fruit number and size [2]. In this respect, observations made during this study further supported the notion that fruit size in this species is a highly plastic trait [21].

The mineral component was highest in the pulp of both species. Although significantly higher in *C. radicans*, mineral content in these fruits was similar to the levels reported previously in other cucurbit fruits [16]. In addition, the N-free extract content was most abundant in pulp, and significantly higher in *C. foetidissima.* This suggests that the pulp of these species contains sizeable amounts of soluble and insoluble carbohydrates, as supported by the high content of NSCs, predominantly Glc, detected in their fruit pulp. In addition to Glc, significantly higher levels of Suc and starch were detected in the pulp of *C. foetidissima*. Other minor components, such as pectins, resins, water soluble organic acids and vitamins, could be also abundant in the peel of these fruits [35]. Additional results indicated that the peels of these fruits were highly fibrosus, containing sizeable contents of both soluble and insoluble fiber. Thus, cellulose and lignin levels were high, as well as ADF, significantly so in *C. foetidissima*. The latter was indicative of important amounts of acid-labile carbohydrates, comprised mostly soluble non-structural carbohydrates, as shown by the NSCs contents detected in the fruit peels of both species. These were almost as high as those detected in the pulp, thereby suggesting that this fruit component may be a digestible source of energy. Additionally, the seed crude fiber content, although higher in *C. foetidissima*, was congruent with that reported previously [25,37]. Contradictory opinions have been voiced regarding high crude fiber content in seeds, arguing, on one hand, that high seed fiber content may be toxic to the digestive process of monogastric animals [16], while, on the other, this same parameter may represent a good source of dietetic fiber for animal nutrition [32].

The seeds of these two wild fruits were a rich of source of both lipids and protein, significantly so in *C. foetidissima* and *C. radicans*, respectively. This property was in accordance with previous reports highlighting the abundance of these nutritional components in the seeds of *C. foetidissima*, which were reported to have high contents of seed oil and protein and be rich in unsaturated fatty acids [16,50] and globulins [18,26,51,52], respectively, the latter of which surpassed 60% of the total seed composition [2,18,22,25,31,37]. This property was similar to that present in seeds of cultivated *Cucurbita* species [32,33,34,35].

HPTLC traces shown in Figure 1 were in accordance with the high neutral lipid content in seeds represented principally by TAGs, with lower content of DAGs and MAGs. These lipid components were also abundant in the fruit pulp and peel of both species, which was contrary to other data showing that these fruit parts usually have a relatively low content of lipids (i.e., 0.04% to 6.57%) [35]. The strong reaction with iodine fumes was in accordance with the abundance of unsaturated fatty acids, mostly linoleic acid (49.63% to 78.82%), followed by palmitic, stearic and oleic acids, present in the different acylglycerols of cucurbit seeds [34,53]. They also agreed with the MALDI-TOF data in which these fatty acids were prevalent in the TAGs and DAGs detected. However, one important difference between the seed oils of *C. radicans* and *C. foetidissima* was the presence of a unique glyceryl trilinolenate in the latter, in addition to other species-specific and unidentified TAGs and DAGS. Another characteristic of the seed oils of these species was that their particular MALDI-TOF patterns, having well-defined and almost equally intense DAG and TAG regions, were closer to those reported in cacao, canola and grape than to soybean, linseed and corn oils [54]. Bands with Rf values different from TAGs, DAGs and MAGs, particularly in pulp and peel extracts suggested the presence of other unsaturated compounds such as carotenes, phytosterols and phospholipids. The presence of waxes in the pulp and peels of fruits of both species was also suggested by strong bands migrating close to the solvent front, whereas abundant α-tocopherol and β-carotenes has also been recorded in the fruit pulps and/or peels of domesticated *C. moschata*, *C. pepo* and *C. maxima* [35,55,56,57]. Plant waxes consist of homologous series of very-long-chain fatty acids, aldehydes, primary and secondary alcohols, ketones, alkanes and alkyl esters, in addition to triterpenoids, tocopherols or aromatic compounds at levels that vary depending on the plant species [58]. Fruit cuticular waxes are necessary to reduce the damage caused by environmental stress and to sustain growth within a relatively stable internal environment. Similar to other fruits, such as apples, they could be key factors in the development, storage and ambient adaptation of these wild cucurbit fruits [59]. Some of the neutral lipid bands detected, mostly in seed extracts, could have represented sterol compounds such as Δ7.22.25-stigmastatrienol, β-sitosterol, spinasterol and Δ7.25-stigmastadienol known to be abundant in other *Cucurbita* fruits [60,61]. Moreover, the presence of phospholipids suggested by the slowly migrating bands in the TLC traces was in agreement with a study reporting that phosphatidylcholine, phosphatidyl-ethanolamine and phosphatidylinositol were the major component of seed linoleic- and myristic acid-rich phospholipids in three species of xerophytic cucurbits, including *C. foetidissima* [62].

The results shown in Figure 2 suggest the presence of non-polar saponins/alkaloids in the pulp and peel of *C. foetidissima* and *C. radicans* fruits. These compounds were more abundant and varied in the fruit parts of the latter species. The emission of fluorescence under UV light that characterizes many fluorescing phytochemicals, such as aromatic alkaloids and tetracyclic isoprenoids having conjugated ring systems in which electron movement, or de-localization, between bonds is possible, supports the proposal that the major fluorescent bands mentioned above could correspond to yet to be defined lipophilic alkaloids similar to the quinolone alkaloids detected in *Citrullus colocynthis*, a medicinal Cucurbitaceae annual plant [63]. Another possibility is that they could represent one or more compounds resembling foetidissimoside A, a 3,28-bidesmosidic triterpenoid saponin previously reported in C. *foetidissima* roots [64,65].

The presence of several cucurbitacins is suggested by data shown in Figure 3 and Table 4. These compounds are triterpenoids with a bitter taste and a toxic nature that are characteristic of Cucurbitaceae [66]. The inferred presence of cucurbitacins B, D, E and I in the pulp and peel of *C. foetidissima* and *C. radicans* fruits is supported by their mobility on the TLC plates and by their fluorescence under UV light, similar to various other reports that have described them in the fruits of several *Cucurbita* species, in which more than 18 cucurbitacin types have been identified [35]. Additionally, the predominant slow-migrating bands (i.e., Rf values ≤ 0.3) were putatively identified as glycosylated cucurbitacins, as reported previously [44,66,67].

The detection of blue and yellow-orange bands was localized in the 0.1–to–0.4 Rf range where the rutin and chlorogenic acid standards were detected. Yellow-orange bands occurred exclusively in fruit peel extracts of both species, but more prominently in *C. foetidissima*. They resembled data from previous HPTLC analysis of pharmaceutically relevant plants [68], in which these bands were tentatively identified as flavonoid quercetin glycosides [69,70,71]. Phenolic compounds were most abundant in peels, whereas the band patterns of seed and pulp extracts were more similar. Based on these and other studies [72,73], the more prominent blue bands could correspond to phenolic acids, such as p-coumaric, ferulic and/or of p-hydroxybenzoic acids, in addition to coumarin. These results contradicted those reported in *C. moschata*, where no flavonoids were detected in the fruit flesh or seeds [74]. However, the proposed presence of flavonols, i.e., quercetine, kaempferol, rutin and quercetin O-glucoside, in addition to phenolic acids, i.e., p-coumaric, p-hydroxybenzoic and coumaric acids coincided with studies describing their abundance in fruit extracts, seeds and peels of diverse *Cucurbita* plants [75,76,77,78,79,80,81]. The identity of the rest of the bands observed in fruit extracts of *C. radicans* and *C. foetidissima* could represent other phenolics such as tyrosol, luteolin, ferulic, caffeic and vanillic acid, vanillin, salicin, stigmast-7,2,2-dien-3-ol, stigmast-7-en-3-ol, p-hydroxybenzaldehyde and protocatechuic acids as reported previously [35].

The electrophoretic analysis of seed proteins of both *Cucurbita* species showed evident differences between them. Thus, the seed protein profile of *C. foetidissima* was more similar to those previously reported in other cucurbit seeds, mostly from domesticated species, in which cucurbitin, an 11S globulin, represents the main storage protein. Cucurbitin is a globular protein consisting of six 54 kDa subunits, each constituted by disulfide-bonded acidic and basic 33 kDa and 22 kDa subunits, respectively, one of which was identified as a 2S albumin with ribonuclease activity [82,83]. Another major band with a molecular weight of 12 kDa was similarly reported in melon (*Colocynthis citrullus*) seed proteins [84]. Bands within this mass range were detected in the seed protein extracts of *C. foetidissima* but not in *C. radicans*. An interesting observation derived from the present study was that the abundance of this protein fraction in seeds of *C. foetidissima* was negatively influenced by the degree of sunlight to which the plants were exposed due to their opposing roadside orientation [21]. This suggests that light could be a factor influencing seed protein composition in this cucurbit plant and perhaps other species. In this respect, increased light conditions during plant growth were found to strongly influence seed size, seed yield and oil content in rapeseed and *Arabidospsis thaliana* via an increased C:N ratio that preferentially enhanced oil over protein storage [85,86]. Other ambient conditions, such as drought and/or high temperatures, have also been found to affect seed protein composition in wheat, lentils, quinoa and other crops [87,88]. Moreover, SDS-PAGE seed protein band patterns in *C. foetidissima* had similarities with *C. moschata* seed proteins, where several other globulins or their proglobulin precursors were detected (i.e. 6.5; 12.82, 16.43, 17.49, 24.61, 32.57 and 56.93 kDa) and with protein band patterns (i.e., major bands at 60.6, 36.8, 44.3 kDa, and between 21.9 and 23.5 kDa, in addition to minor bands in the range of 7−10 kDa) reported in pumpkin seed proteins [45,48,89,90].

Glutelins are also among the principal cucurbit seed proteins while albumins and prolamins are present in lower abundance. This contrasted with the protein band patterns produced by *C. radicans* seed extracts, where the prominent globulin bands were greatly diminished and were replaced by bands whose migration corresponded to putative glutelin and albumin proteins [91,92,93]. This variation was in accordance with the seed protein seed composition of bitter melon (*Momordica charantia*), which was constituted mostly by albumins and globulins [94] and agreed with a battery of studies showing different species-specific protein profiles within the Cucurbitaceae family [92].

The detection of low levels of NSCs and RFOs in *C. foetidissima* and *C. radicans* fruit parts was in agreement with various studies reporting reduced amounts Glc and Suc as well as the Raf and Sta RFOs in Cucurbit fruits, such as *C. maxima* [95,96,97,98,99]. Starch accumulated in very low amounts, as expected [100], while Glc and Fru were the predominant sugars in the fruit pulp of the two wild *Cucurbita* fruits. Glc was found, however, to be unusually high in the peels of *C. foetidissima* fruits, which was ca. 3-fold higher than the levels detected in *C. radicans* fruit peels. However, soluble NSC levels were 2- to 4-fold lower than those reported previously in wild and domesticated *Cucurbita* fruits, including *C. foetidissima* [101]. The Raf and Sta content reported in this study was also much lower than that reported in other cucurbit fruits, particularly in those of xerophyte species, where Raf was found to accumulate to higher levels (i.e., 1.1–1.2%) than in domesticated ones (i.e., 0.6–0.8%), whereas no verbascose was detected [101,102]. However, an unidentified compound with a retention time similar to that produced by the trehalose disaccharide was detected instead. Another peculiarity of the peel and pulp of *C. foetidissima* and *C. radicans* fruits was the detection of the RFO precursors MI and GOL at levels that were higher than the RFOs themselves. The low RFO content observed agreed with studies showing that these potentially anti-nutritional factors are much lower than in commercial leguminous seeds, such as soybean. An interesting outcome was that except for Fru in the pulp, MI in the seeds and peels and Sta in the peels of *C. radicans*, the levels of almost all NSCs and RFOs analyzed were higher in *C. foetidissima*. This result suggested species-specific differences in carbon partitioning and in the synthesis of RFOs, acting perhaps as abiotic stress-regulating osmolytes and/or seed vigor/seed-desiccation tolerance factors, as reported previously in cucurbits and other plant species [103,104,105,106,107,108,109].

The variations in the fruit composition observed between the two *Cucurbita* wild species probably reflected the notion regarding *C. foetidissima* as an aberrant species having no affinity with any other *Cucurbita* species [110]. Other contributing factors could have been the following: (i) different climatic conditions encountered in the sampling sites, mostly rainfall levels, which have been reported to influence the chemical composition of pumpkin seeds [111] and the plant morphology and yield of *C. foetidissima* [3]; (ii) the known genetic variation, at least within *C. foetidissima* plants, that shows a wide range of plastic adaptations to changeable environmental conditions [2] and (iii) the type and abundance of competing species growing in the sites.

The metabolic and phenotypic shown by these wild *Cucurbita* species was in agreement with a common plant survival strategy by means of which plants from the same genotype but growing in different environments are able to adopt different life-history strategies to maximize resource allocation [112]. For instance, the phenotypic and metabolic plasticity shown by *Brachypodium distachyon* plants under different stress combinations indicated that this trait strongly defined their acclimation and possible geographical dispersion under changing environments [113]. This study emphasized the importance of understanding how the limits of plasticity under changing environments may allow the prediction of species dispersion and facilitate the development of varieties better adapted to varying climatic conditions.

In this sense, the proposed metabolic flexibility could offer solutions to a highly probable future scenario in which wild and domesticated *Cucurbita* species will face increasingly demanding challenges as climate change intensifies [114]. Among the predicted risks among cultivated cucurbits are an increased prevalence of and higher susceptibility to pathogen infection and subsequent disease development [115], and augmented fruit putrefaction due to changes in precipitation on one side, and reduced yields due extended drought on the other [116]. However, similar to projections based on the results of a model devised to determine the effects of climate change on the distribution of *Tagetes lucida*, an endemic Mexican species [117], these benefits could be greatly compromised by future climate change scenarios leading to decreased populations of wild *Cucurbita* species due to habitat contraction, fragmentation or loss [114]. More focused and extended efforts will be needed to fully determine the chemical composition of the fruits of these marginally studied fruits and the effect on their phytochemistry exerted by the ever more fluctuating ambient conditions caused by the inevitable global warming syndrome, particularly so in the vulnerable arid and semi-arid regions of Mexico [43].

## 4. Materials and Methods

Study sites: *Cucurbita foetidissima* and *C. radicans* fruits were sampled in different locations situated in the state of Jalisco, México: the former, in the localities of Vaquerías, in the municipality of Ojuelos (21°47’28.39” N and 101°36’7.03” W) and the latter in San José de Gracia, in the municipality of Tepatitlán de Morelos (20° 41’ 8.16”N and 102° 34’ 11.42” W). Vaquerías is localized at 2222 m a.s.l. Its mean annual rainfall precipitation, ranging between 424 and 450 mm according to a 30-year historical record of this region, corresponds to a semi-arid region [118]. The San José de Gracia location is higher, at 1947 m a.s.l., and more humid, with a mean annual rainfall precipitation ranging between 900 and 1000 mm.

Sampling design and morphological assessment: The study sites were delimited in 2017 while fruit sampling was performed in the autumn of 2018. Four to ten fruits per plant were sampled from individual *C. foetidissima* and *C. radicans* plants selected randomly. The sampled *C. foetidissima* plants were growing along the roadside of a two-lane paved highway. The sampling approach integrated five plants per roadside verge, which were oriented to the East and West, respectively. *Cucurbita radicans* fruits were collected from plants growing in grasslands, vacant lots and/or the edge of rural roads, usually in association with *Prosopis* sp. and other leguminous shrubs [21]. Considering that fruit maturation in *C. foetidissima* includes two well-defined stages, care was taken to sample fruits that had already reached full maturity. This maturation stage is characterized by a homogenous pale-yellow coloration of the fruit skin and full seed development [23]. Fruits of *C. radicans* were collected while still immature and were allowed to mature at room temperature on laboratory benches or in desiccators, taking care to avoid fungal infection. Mature fruits of both species were separated into their respective seed, pulp and peel sections. Except for proximal composition analyses for which the fruit sections were dried in an oven until reaching constant weight and then ground to a fine powder (see below), pulp and peels were flash-frozen with liquid nitrogen and stored at -70 °C until needed for analysis, whereas the seeds were kept in a desiccator. All fruits were placed in sackcloth bags and were immediately transported to the Laboratorio de Ecología Fitoquímica y Molecular; Centro Universitario de Ciencias Biológicas y Agropecuarias (CUCBA), Universidad de Guadalajara, Mexico, for subsequent analysis. Fruit specimens of *C. foetidissima* and *C. radicans* were also sent to the Luz María Villareal de Puga herbarium of the Universidad de Guadalajara (IBUG) and were registered as exhibit No. 211690 (*C. foetidissima*) and No. 211688 (*C. radicans*) for taxonomic identification purposes.

Morphological variables of intact fruits that were analyzed were the following: fruit fresh weight (FFW) and equatorial (ED) and longitudinal (LD) diameters, respectively. Fruits were weighed using a Pioneer analytical balance (OHAUS Latin America, Mexico City, Mexico). The fruit’s ED and LD were recorded, in cm, employing a digital Vernier caliper (Mitutoyo Absolute Digital Caliper; Mitutoyo South Asia Pvt. Ltd., Kanagawa, Japan). Fruit tissues were subsequently divided into peel, pulp and seed. The weight of total seeds per fruit (SW), of 100 seeds/fruit (W100S) and of their respective peel (PeFW) and pulp (PuFW) was registered using the same analytical balance. Also recorded were the total number of seeds (SN) per fruit.

Proximate Analysis: Prior to analysis, the fruit components were divided into pulp, peel and seeds. These were dried to constant weight at 70 °C for 72 h. The dried materials were pulverized and homogenized. Ten-gram samples were placed in plastic bags until sent for analysis at the Laboratorio de Nutrición Animal de la División de Ciencias Veterinarias, Universidad de Guadalajara, México. The analyses were performed according to standard AOAC methods [119]: crude protein (N × 6.25; AOAC 954.01), total lipid content (AOAC 920.39), ash (AOAC 942.05), dry matter (AOAC 934.01) and crude fiber (AOAC 954.01). Additionally, the AOAC 973.18-1977 method was employed for the gravimetric determination of digestible acid detergent fiber (ADF), lignin and cellulose. Two replicate analyses were performed, using pooled samples consisting of 10 combined fruits taken from 5 plants.

Extraction of lipid fraction: Lipids were extracted from lyophilized and finely pulverized pulp, seed and peel sections of *C. foetidissima* and *C. radicans* fruits. Five grams were extracted with 100 mL hexane using a Büchi Soxhlet Extraction Unit (E-816 SOX; BÜCHI Labortechnik, Postfach, CH) for 10 cycles at 70 °C during 1.15 h. The resulting extracts were evaporated to dryness under vacuum and the total lipid content was determined based on the apparent density of sunflower oil (0.9205 g/cm^3^). Part of the extracts were re-dissolved in 4 mL of a CHCl_3_-CH_3_OH (2:1 *v/v*) solution for the subsequent high-performance thin layer chromatography (HPTLC) analysis of neutral lipids (see below).

Thin layer chromatography of fruit seed, pulp and peel extracts: Pulp, peel and seed fruit tissues of both species were analyzed by HPTLC for the determination of different chemical fractions. The analyses were performed using 20 × 10 cm HPTLC silica 60 F254 gel plates (Merck KGaA, Darmstadt, Germany). The samples were applied using a CAMAG automatic ATS4 TLC sampler (CAMAG Chemie-Erzeugnisse & Adsorptionstechnik AG, Muttenz, Switzerland) and were separated using the appropriate mobile phases and developing solutions or strategies. Plates were developed in a CAMAG ADC2 automatic developing chamber. Neutral lipids were analyzed by applying 10 μL (seed and standards) or 30 μL (peel and pulp) of 10 mg/mL chloroform: methanol 2:1 *v/v* extracts (obtained from initial hexane extracts; see above) on the silica TLC plates that were developed for 20 min at 4 °C using an 80:20:1 *v/v/v* petroleum ether: ethyl ether: acetic acid mobile phase. The 8.0 mm wide tracks, placed 15.0 mm away from the lateral and lower edges, were used to develop the different *Cucurbita* fruit samples, plus olive and/or sunflower samples and the cholesterol, stigmasterol and stearic acid standards, also at 10 mg/mL, used for comparison and identification. The plates were developed in the glass chamber previously saturated for 20 min with iodine fumes. Visualization and documentation were performed with a CAMAG TLC visualizer under white light, after iodine development. The neutral lipid bands were classified by comparing their Rf values with those reported for known lipid standards. Saponins (e.g., cucurbitacins) were determined according to previously reported procedures [69,120]. Briefly, 5 mg of lyophilized fruit tissues were dissolved in 100 mL absolute ethanol. The EtOH extracts (at 1 mg/mL) were subsequently dissolved in 1 mL of CHCl_3_-CH_3_OH (1:1 *v/v*) solution. Five μL of the respective extracts were applied on to the HPTLC plates which were developed using a 95:10 *v/v* chloroform: methanol mobile phase. The plates were visualized under UV light (254 nm) and after derivatization with a vanillin: phosphoric acid: ethanol (80:20:1 *v/v/v*) solution. Bands became visible after heating the plates for 10 min. A previously reported method [121] was used for the analysis of phenolic compounds. Thus, 100 mg of lyophilized fruit tissues were dissolved in 1 mL of 80% methanol and 10 μL aliquots of each were placed on the plates which were developed with a 50:5:5:7.5 *v/v/v/v* ethyl acetate: formic acid: acetic acid: water mobile phase in the ADC2 developing chamber previously saturated for 20 min with a potassium thiocyanate solution, and conditioned at 47% relative humidity for 10 min. For post-separation derivatization, the plates were heated at 100 °C for 3 min and, while still hot, immersed into a Natural Products A reagent (Carl Roth GmbH + Co. KG; Karlsruhe, Germany), dried and subsequently dipped into a polyethylene glycol 400 solution. In both cases, immersion speed and duration were 3 cm/s and 3s, respectively. Documentation was performed under UV light (at 366 nm) after derivatization. Results were compared to chlorogenic acid and rutin standards.

Protein content and SDS-PAGE separation: One g of lyophilized pulp, peel and seed tissues, respectively, were dissolved by vortexing in 1 mL of phosphate buffer, pH 7.2 (Gibco; Thermo-Fisher Scientific; Waltham, MA, USA) containing a proteinase inhibitor cocktail (Merck KGaA, Darmstadt, Germany). The extracts were centrifuged twice, first at 4000× *g* (8 min at 4 °C) and then at 12000× *g* (10 min a 4 °C). The protein content of the cleared supernatants was determined according to the Bradford method [122] using a commercial kit (Bio-Rad, Hercules, CA, USA) and bovine serum albumin as standard. The SDS-PAGE seed protein profiles were determined by dissolving 30 μg of lyophilized tissue in 20 μL of 4 × sampling buffer + water. The protein solutions were denatured by incubating in boiling water for 5 min prior to the SDS-PAGE separation, which was performed on 10% polyacrylamide gels run at 100 V for 2 h. The gels were stained with a blue Coomassie Blue R-250 solution, for 5 min, and subsequently de-stained using a methanol: acetic acid 250:37.5 *v/v* solution. The developed protein bands were visualized using a commercial gel documentation system (ChemiDoc Imaging Systems; Bio-Rad). They were classified according to their molecular mass, as per the molecular mass markers [45,46,47,48,123].

Quantitation of non-structural carbohydrates (NSCs) and raffinose family oligosaccharides (RFOs): Soluble (i.e., glucose (Glc), fructose (Fru) and sucrose (Suc)) and insoluble (i.e., starch) NSCs were determined in fruit section extracts obtained from mixing 20 mg of lyophilized ground fruit tissues in 0.6 mL of extraction buffer (50 mM Hepes KOH, pH 7.4, and 5 mM MgCl_2_ in 80% ethanol). NSCs were determined enzymatically using a coupled assay with glucose-6-phosphate dehydrogenase (from yeast, grade II, Roche, Mannheim, Germany) in which NADPH formation was measured at 340 nm [124]. For starch determination, the pellets resulting from the preparation of the soluble NSC extracts were resuspended in 0.5 mL of 10 mM KOH and autoclaved for 30 min. Starch was hydrolyzed overnight at 37 °C in 0.7 mL of starch degradation buffer (100 mM Hepes, pH 7.5, 3 mM MgCl_2_, 10 U of amylase (α-amylase), Type VI-B, from porcine pancreas (E.C. number 3.2.1.1; Sigma-Aldrich Chemical, St. Louis MO, USA) and 10 U of amyloglucosidase from Aspergillus niger (E.C. number 3.2.1.3; Sigma)). After centrifugation at 2900× *g*, the supernatants were stored at 4 °C. The pellet was re-extracted with 0.5 mL of starch degradation buffer at 37 °C for 30 min. After centrifugation, the supernatants were combined and assayed enzymatically for Glc as described above. Identification and determination of RFOs content in different fruit section extracts were performed by High-Performance Anion-Exchange Chromatography with Pulsed Amperometric Detection, as described previously [125]. All chemicals used to standardize the chromatographic separation and for RFOs quantitation were from Sigma (Sigma-Aldrich Chemical, St. Louis, MO, USA). RFOs quantified were: Myo-inositol (MI), galactinol (GOL), raffinose (RAF) and staquiose (STA). A trehalose-like compound was also determined using this method.

Characterization of seed oils by direct matrix-assisted direct laser desorption/ionization time-of-flight mass spectroscopy (MALDI-TOF): Seeds (3 g) were extracted with 100 mL of chromatography level hexane (Karal, S.A. de C.V.; León. Gto., México) in a Büchi extraction unit, using ten evaporation-condensation cycles at 70 °C (ca. 75 min), followed by 15 min and 30 min washing and drying steps, respectively. The dried hexane extract was re-extracted with a 1:1 *v/v* chloroform: methanol solution that was adjusted to a concentration of 1 mg/mL. A MALDI matrix solution was prepared by dissolving 2,5-dihydroxybenzoic acid in 100% acetonitrile at 20 mg/mL. Small volumes of samples (0.7 μL) and matrix (1 μL) were mixed together, and 1 μL was then applied directly to a stainless steel MALDI target. An accelerating voltage of 20 kV was used. The MALDI-TOF MS spectra were recorded in reflectron mode within a mass range of m/z 450-2400 using an AutoFlex III mass spectrometer (Bruker Daltonics; Bruker; Billerica, MA, USA) [126].

Data analysis. The study had a factorial design. “Species” was the first factor with two levels: *Cucurbita foetidissima* and *C. radicans*. “Fruit part” was the second factor, in which three levels were established: seed, pulp and peel. For analysis, *C. foetidissima* and *C. radicans* fruits were combined into 5- and 2-fruit pools, respectively. NSCs and RFOs variables were analyzed via two-way ANOVAs followed by Tukey HSD tests (*p* ≤ 0.05) using the SigmaPlot 11.0 package.

## Figures and Tables

**Table 1:** Morphological characteristics of *Cucurbita foetidissima* and *C. radicans* fruits. Morphological variables were measured in fruits of *C. foetidissima* and *C. radicans* plants collected in the wild from sites localized in the state of Jalisco, Mexico.

	FFW ^3^	ED	LD	SW	TSN	W100S	PuFW	PeFW
*C. radicans* ^1^	90.3 ± 19.8	6.4 ± 0.4	5.5 ± 0.3	7.0 ± 2.2	93 ± 30.9	7.0 ± 1.8 *	55.6 ± 10.2	23.9 ± 2.9
*C. foetidissima* ^1^	148.8 ± 21.4 *^,2^	6.7 ± 0.3	6.7 ± 0.2 *	16.5 ± 1.1 *	280 ± 18.5 *	6.1 ± 0.9	80.2 ± 12.3 *	35.1 ± 9.5 *

^1^*C. radicans* and *C. foetidissima* were sampled in the year 2018 in the communities of San José de Gracia and Vaquerías, Jalisco, México, respectively. ^2^ Asterisks (*) indicate statistically significant pair-wise differences (Anova, Tukey HSD tests [*p* ≤ 0.05]). ^3^ ED = Equatorial diameter; FFW = Fruit fresh weight; LD = Longitudinal diameter; PeFW = Peel fresh weight; PuFW = Pulp fresh weight; SN = Total seed number; SW = Fruit seed weight; W100S = Weight of 100 seeds.

**Table 2:** Proximal composition in *Cucurbita foetidissima* and *C. radicans* fruits. Proximal composition variables were measured in fruits of *C. foetidissima* and *C. radicans* plants collected in the wild from sites localized in the state of Jalisco, Mexico.

	PEEL		SEED		PULP	
%	*C. radicans* ^1^	*C. foetidissima* ^1^	*C. radicans*	*C. foetidissima*	*C. radicans*	*C. foetidissima*
Dry Matter	95.3 ± 3.1	95.6 ± 3.8	95.9 ± 2.8	96.6 ± 2.2	91.4 ± 4.7	93.2 ± 5.1
Ash (minerals)	6.1 ± 1.0	7.0 ± 0.8	5.2 ± 0.7	4.4 ± 0.7	16.1 ± 0.6 *^,4^	15.9 ± 1.0
Crude Protein	9.8 ± 4.0	6.6 ± 1.3	33.1 ± 3.0 *	30.6 ± 2.9	18.2 ± 2.4	13.4 ± 4.3
Total Lipids	2.17 ± 2.0	2.5 ± 2.5	28.7 ± 4.1	32.3 ± 1.3 *	3.3 ± 4.3	0.89 ± 0.5
Total Fiber	42.1 ± 3.8	48.4 ± 5.5 *	21.8 ± 1.9	26.5 ± 1.8	19.7 ± 3.9	19.0 ± 1.7
N-free Extract ^2^	34.2 ± 10.8 *	31.0 ± 3.2	8.6 ± 6.0	4.1 ± 4.7	34.0 ± 10.8	44.1 ± 2.9 *
ADF ^3^	51.9 ± 3.8	55.9 ± 8.1 *	36.6 ± 4.8	38.3 ± 5.4 *	29.0 ± 6.0	29.9 ± 4.3
Lignin	16.5 ± 2.3	18.7 ± 4.2 *	15.3 ± 2.0	17.8 ± 5.3 *	4.5 ± 3.8	2.6 ± 0.7
Cellulose ^2^	35.4 ± 2.0	37.2 ± 4.1 *	21.3 ± 3.3	20.2 ± 1.1	24.5 ± 3.6	27.3 ± 4.3

^1^*C. radicans* and *C. foetidissima* were sampled in the year 2018 in the communities of San José de Gracia and Vaquerías, Jalisco, México, respectively. ^2^ Calculated by difference. ^3^ ADF = Acid Detergent Fiber. ^4^ Asterisks (*) indicate statistically significant pair-wise difference (Anova, Tukey HSD tests [*p* ≤ 0.05]).

**Table 3:** Fatty acid composition of *Cucurbita* seed oils. The composition of the oils extracted from seeds of *C. radicans* and *C. foetidissima* was determined by MALDI-TOF. The identity of the most relevant m/z ions was determined on the basis of the standards shown and on published data.

Standards (m/z)	ID	*C. radicans* ^1^	ID	*C. foetidissima* ^1^	ID
550.673	Matrix peak?				
				551.251	NI
		567.247	NI ^2^		
		599.827	PO (DAG) ^3^	599.84	PO (DAG)
601.6	PS				
638.671	LaLaLa				
				657.841	NI
661.611	LaLaLa (Na^+^)				
696.713					
745.708	MMM (Na^+^)				
				759.067	NI
761.681	MMM (K^+^)				
				783.097	NI
				805.096	NI
829.8	PPP (Na^+^)				
		878.341	PLL (TAG)	878.353	PLL
		880.348	POL	880.368	POL
		892.243	NI		
		894.325	NI	894.339	NI
895.756	LnLnLn (Na^+^)	896.323	LnLnLn		
901.806	LLL (Na^+^)	902.374	LLL	902.374	LLL
		*904.382*	LLO	904.394	LLO
		906.394	OOL	906.42	OOL
907.855	OOO (Na^+^)				
				908.43	OOS
911.755	SOS-SSO (Na^+^)				
913.897	SSS (Na^+^)				
		916.35	NI		
917.794	LLL (K^+^)				
		918.36	NI	918.317	NI
		920.367	NI	920.38	NI
		922.373	NI	922.4	NI
		934.36	NI		
943.742	NI				

^1^*C. radicans* and *C. foetidissima* were sampled in the year 2018 in the communities of San José de Gracia and Vaquerías, Jalisco, México, respectively. ^2^ NI = Not identified. ^3^ DAG = diacylglycerol; TAG = triacylglycerol; M = myristic acid; P = palmitic acid; S = stearic acid; O = oleic acid; L = linoleic acid; Ln = linolenic acid.

**Table 4:** Identification of putative cucurbitacins in fruits of wild *Cucurbita foetidissima* and *C. radicans* fruits collected in the wild ^1^. The retention factors (Rf) were obtained from bands derived from the HPTLC separation of pulp, seed and peel ethanol extracts of *C. foetidissima* and *C. radicans* fruits collected from plants growing in the wild in sites localized in the state of Jalisco, México. The Rf values were compared to those reported by Ríos et al. [44].

Cucurbitacin Glycosides	Rf Value ^2^ Range	Non-Glycosylated Cucurbitacins	Rf Value Range
Cucurbitacin-L-glucoside Cucurbitacin-I-glucoside	0.14–0.18	Cucurbitacin D Cucurbitacin C Cucurbitacin A Cucurbitacin L	0.58–0.69
Cucurbitacin-B-glucoside Cucurbitacin-E-glucoside 23, 24 dihydrocucurbitacin E-glucoside	0.26–0.30		
		Cucurbitacin I Cucurbitacin B 23, 24-dihydrocucurbitacin B 23, 24-dihydrocucurbitacin E Cucurbitacin E	0.79–0.95

^1^*C. radicans* (*Cr*) and *C. foetidissima* (*Cf*) were sampled in the year 2018 in the communities of San José de Gracia (*Cr*) and Vaquerías (*Cf*), Jalisco, México (municipalities of Tepatitlán and Ojuelos, respectively). ^2^ The Rf value- range shown is representative of the bands shown in Figure 3.

**Table 5:** Soluble and insoluble non-structural carbohydrate (NSCs) levels in *Cucurbita foetidissima* and *C. radicans* fruits. NSC content was determined in fruits collected from *C. foetidissima* and *C. radicans* plants collected in the wild from sites localized in the state of Jalisco, Mexico.

	SEED		PULP		PEEL	
mg/g (FWB) ^2^	*C. foetidissima* ^1^	*C. radicans*	*C. foetidissima*	*C. radicans*	*C. foetidissima*	*C. radicans*
Glucose	1.97 ± 0.01 **^,3^	1.4 ± 0.02	20.7 ± 0.2 **	17.8 ± 0.2	19.6 ± 0.2 **	7.18 ± 0.1
Fructose	1.98 ± 0.03 **	1.32 ± 0.03	12.8 ± 0.08	14.9 ± 0.04 **	12.4 ± 0.2 **	10.1 ± 0.13
Sucrose	8.28 ± 0.08	8.04 ± 0.08	5.16 ± 0.2 **	2.63 ± 0.08	2.97 ± 0.05	3.11 ± 0.07
Starch	0.33 ± 0.04 *	0.14 ± 0.02	5.57 ± 0.13 **	4.29 ± 0.2	3.12 ± 0.14 **	0.58 ± 0.05

^1^*C. radicans* and *C. foetidissima* were sampled in the year 2018 in the communities of San José de Gracia and Vaquerías, Jalisco, Mexico, respectively. ^2^ FWB = Fresh Weight Basis. ^3^ Asterisks indicate statistically significant pair-wise differences (Anova, Tukey HSD tests, at *p* ˂ 0.05 * or *p ˂* 0.01 **).

**Table 6:** Determination of raffinose family oligosaccharides (RFOs) in *Cucurbita foetidissima* and *C. radicans* fruits collected in the wild. RFO levels were measured in fruits collected from *C. foetidissima* and *C. radicans* plants collected in the wild from sites localized in the state of Jalisco, Mexico.

	SEED		PULP		PEEL	
mg/g (DWB) ^2^	*C. foetidissima* ^1^	*C. radicans*	*C. foetidissima*	*C. radicans*	*C. foetidissima*	*C. radicans*
Myo-inositol	1.02 ± 0.01	1.31 ± 0.04 **	3.52 ± 0.16	4.48 ± 0.48	2.38 ± 0.02	5.46 ± 0.08 **
Galactinol	0.84 ± 0.02 *	0.67 ± 0.02	2.99 ± 0.31	2.41 ± 0.39	3.36 ± 0.15	3.17 ± 0.12
NI-RFO ^4^	0.40 ± 0.02	0.43 ± 0.04	1.93 ± 0.27 *	0.90 ± 0.18	1.21 ± 0.09	1.18 ± 0.03
Raffinose	1.85 ± 0.06 *^,3^	1.54 ± 0.05	0.10 ± 0.02	0.06 ± 0.01	0.35 ± 0.01 **	0.11 ± 0.01
Staquiose	4.73 ± 0.1 *	3.74 ± 0.21	0.62 ± 0.07	0.75 ± 0.1	0.19 ± 0.01	0.55 ± 0.08 **

^1^*C. radicans* and *C. foetidissima* were sampled in the year 2018 in the communities of San José de Gracia and Vaquerías, Jalisco, Mexico, respectively. ^2^ DWB = Dry Weight Basis. ^3^ Asterisks indicate statistically significant pair-wise differences (Anova, Tukey HSD tests, at *p* ˂ 0.05 * or *p* ˂ 0.01 **). ^4^ NI-RFO = Not identified RFO.

## Data Availability

The data presented in this study are available on request from the corresponding authors.

## Supplementary Materials

The following are available online at www.mdpi.com/article/10.3390/plants10112451/s1, Figure S1: MALDI TOF/MS mass spectra (m/z 550-950 region) of (**a**) *Cucurbita radicans* and (**b**) *C. foetidissima* seed oils.
