# Supplementary material for: Contrasting Metabolic Fingerprints and Seed Protein Profiles of Cucurbita foetidissima and C. radicans Fruits from Feral Plants Sampled in Central Mexico
Source: Plants (Basel). 2021 Nov 13;10(11):2451. doi: 10.3390/plants10112451 (PMC8617929; doi:10.3390/plants10112451)
Supplement: Supplementary file 1 [file plants-10-02451-s001.zip › plants-1418734-supplementary.pdf]

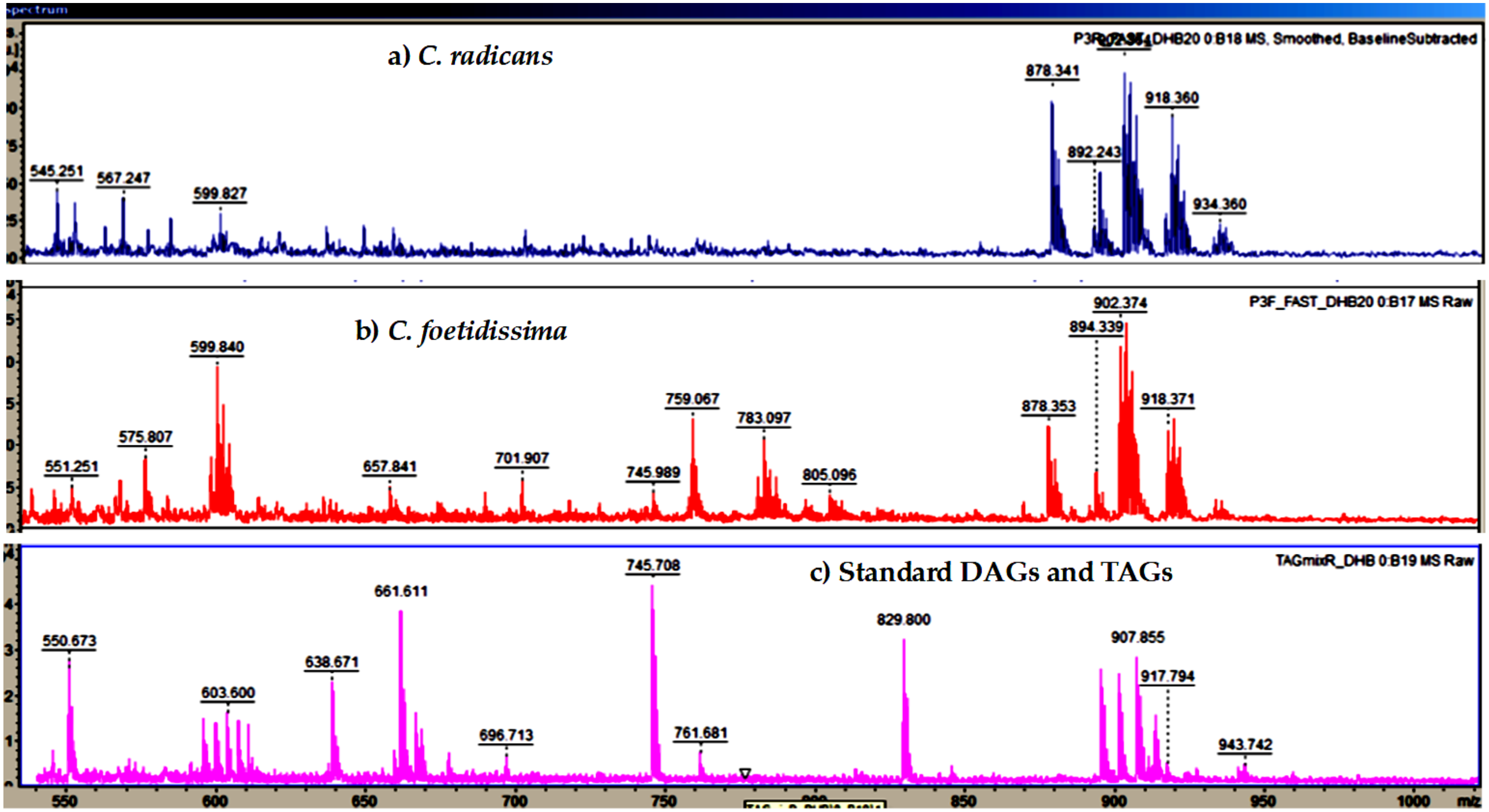

Figure S1. MALDI TOF/MS mass spectra (m/z 550-950 region) of (**a**) *Cucurbita radicans* and (**b**) *C. foetidissima* seed oils. The spectra include both triacylglycerol (TAG) and diacylglycerol (TAG) regions. The spectra were recorded in positive ion mode. Many are sodium ion adducts. In (**c**) TAG and DAG standards are shown.
